# Natural killer cell activity in prostate cancer patients treated with curative radiotherapy with or without androgen deprivation therapy: an observational study

**DOI:** 10.2340/1651-226X.2025.44007

**Published:** 2025-10-19

**Authors:** Stine V. Eriksen, Christine V. Madsen, Signe Timm, Ahmed H. Zedan, Louise Raunkilde, Torben F. Hansen, Line Nederby

**Affiliations:** a Department of Oncology, Vejle Hospital, University Hospital of Southern Denmark, Vejle, Denmark; b Department of Regional Health Research, University of Southern Denmark, Odense, Denmark; c Department of Biochemistry and Immunology, Vejle Hospital, University Hospital of Southern Denmark, Vejle, Denmark

**Keywords:** Natural killer cells, prostate cancer, radiotherapy, androgen deprivation therapy, immune biomarkers

## Abstract

**Background and purpose:**

Natural killer (NK) cells play an important role in defense against cancer. Low NK cell activity (NKA) has been linked to prostate cancer (PCa) detection, and effective NKA may be associated with better prognosis in metastatic PCa. Radiotherapy (RT) could affect the immune response, but data on NKA in patients with PCa receiving RT ± androgen deprivation therapy (ADT) remain limited. Hence, this study investigated NKA in such patients.

**Patient/material and methods:**

Peripheral blood from 150 patients with PCa receiving curatively intended RT was collected into NK Vue^®^ tubes prior to RT (baseline, BL), after end of RT (EOT), and during follow-up. Patients received 0- (*n* = 15), 6- (*n* = 23), or 36-months of ADT (*n* = 112), starting 3 months before RT. Interferon-γ was a surrogate marker for NKA in NK Vue^®^ tubes. Data were analyzed using descriptive statistics.

**Results:**

Baseline characteristics were similar between patients with normal (≥ 250 pg/mL) (*n* = 46) and low (< 250 pg/mL) (*n* = 104) NKA; however, smoking was more prevalent in the low NKA group (28% vs. 11%). The distribution of NKA levels differed between groups and time points, notably showing a decreased interquartile range (IQR) for all groups at EOT (BL median 832 pg/mL, IQR 2901; EOT median 312 pg/mL, IQR 708). NKA fluctuated during follow-up and did not mirror prostate-specific antigen dynamics.

**Interpretation:**

Patients with localized PCa treated with RT ± ADT displayed marked variation in NKA, including treatment-related dynamics. The overall complexity and heterogeneity of NKA raise questions about its clinical utility as a biomarker in this setting.

## Introduction

Natural Killer (NK) cells are cytotoxic lymphocytes that play a crucial role in the innate immune system and contribute to suppressing malignancies and viral infections. Their key antitumor functions include recognizing tumor cells, rapidly producing tumoricidal cytokines and chemokines, mediating cytotoxicity, and recruiting other immune cells [1, 2]. Interferon-γ (IFN-γ) represents a key cytokine in this context, secreted upon the activation of the NK cells and subsequently orchestrating the recruitment and activation of the immune system, while also directly suppressing tumor cell growth [3]. Assessment of a subject’s NK cell capacity to produce IFN-γ is commonly employed in immunological and translational research as a surrogate marker of NK cell activity (NKA). NK cells constitute a heterogeneous population that vary across different tissues [2, 4].

For more than 20 years ago, Imai et al. demonstrated a correlation between reduced activity of peripheral-blood lymphocytes and an increased risk of cancer, underscoring the potential role of innate immune defense mechanisms in protecting against cancer development [5].

Focusing on prostate cancer (PCa) and localized/locally advanced disease, several studies indicate a correlation between low NKA, measured by the concentration of interferon-gamma (IFN-γ) cytokine released in peripheral blood, and the risk of detecting PCa at transrectal prostate biopsies [6–9]. Furthermore, lower IFN-γ levels have been reported preoperatively compared to postoperatively in patients undergoing radical prostatectomy for PCa [10]. The study by Song et al. found no correlation between IFN-γ levels and the detection of PCa nor between IFN-γ and Gleason grade [11], highlighting that our understanding of the relationship between NKA and PCa remains incomplete.

Immunotherapy in PCa has yet to deliver the promising results observed in other tumor types. However, it has been speculated whether ‘warming up’ the ‘cold’ prostate tumor microenvironment by harnessing NKA could be a promising strategy for enhancing its responsiveness to current immunotherapies [12].

Radiotherapy (RT) not only induces cell damage but can also modulate immune activity within the tumor microenvironment, including reducing hypoxia and activating NK cells. Reducing hypoxia is believed to reactivate NK cells [13]. Reoxygenation to increase tumor response is of great interest and has been investigated in some PCa studies. One study demonstrated prostate tumor reoxygenation in intermediate-risk PCa receiving RT without androgen deprivation therapy (ADT) [14], and neoadjuvant ADT itself might also cause reoxygenation in PCa [15]. Moreover, activated NK cells may enhance the tumor’s response to RT by increasing the radiosensitivity [13]. This theoretically synergistic effect has led to the initiation of several preclinical and clinical trials exploring the combination of NK cell therapy and RT for various solid tumors [13]. However, many aspects remain unresolved regarding the interplay between tumor response in different tumors, NK cell activation, reoxygenation, and RT fractionation.

Treatment with ADT lowers testosterone levels, contributes to a reduction in tumor burden, and may modulate immune cell infiltration in patients with PCa [16]. This regulation likely involves effects on T- and B-cell development through testosterone’s interaction with the thymus and bone marrow, respectively [17]. The reduction in testosterone induced by ADT has been associated with an increase in peripheral NKA [18] and enhanced T-cell responsiveness [19]. However, these effects were not observed in the small study conducted by Jochems et al. [20].

In this study, we hypothesized that treatment would affect NKA. Additionally, ADT might independently modulate NKA, potentially reflecting improved tumor control. Persistently low or declining NKA levels could be associated with an increased risk of biochemical recurrence.

## Patients/material and methods

### Study cohort

Between May 2019 and November 2023, 162 patients treated with curatively intended RT for PCa were consecutively included in this biomarker study. Blood samples for biomarker analyses and prostate-specific antigen (PSA) were collected before the start of RT (baseline, denoted BL), at the end of treatment (EOT), and during follow-up at 3, 6, 9, 12, 18, 24, 30, 36, 48, and 60 months. BL samples were collected after the initiation of ADT, if given. Due to missing BL samples, 12 patients were excluded from the analysis. To ensure high representativeness, no other exclusion criteria were applied.

The prescribed dose to the prostate was either 78 Gy in 39 fractions (*n* = 128) or 60 Gy in 20 fractions (*n* = 22). The majority of patients (*n* = 126) received elective lymph node irradiation as well. Based on the European Association of Urology (EAU) risk group classification [21], patients received either no ADT (*n* = 15), 6 months of ADT (*n* = 23), or 36 months of ADT (*n* = 112), starting three months before RT; these are hereafter referred to as the three ADT groups. ADT was administered predominantly using the GnRH agonist leuprorelin acetate, which was used in 97% of cases.

Biochemical recurrence was defined according to Radiation Therapy Oncology Group (RTOG), American Society for Therapeutic Radiology and Oncology (ASTRO) Phoenix Consensus Conference definition [22].

The study protocol was approved by the Regional Committee on Health Research Ethics for Southern Denmark (Project ID: S-20190021) and the Danish Data Protection Agency (ID: 19/11099). Written informed consent was obtained from all patients, and the study adhered strictly to the Helsinki II Declaration.

This study is reported in accordance with the STROBE (Strengthening the Reporting of Observational Studies in Epidemiology) guidelines [23].

### NK Cell Activity Measurement

One milliliter of whole blood was collected into NK Vue^®^ tubes (NKMAX Co., Ltd., South Korea) and incubated at 37°C within 15 min. of collection. After a 24-h incubation period, the concentration of IFN-γ in the plasma was quantified using the NK Vue ELISA (NKMAX Co., Ltd.), serving as a surrogate marker for NKA [24]. Samples exceeding the upper limit of quantification (2,000 pg/mL) were diluted at 1:10 and reanalyzed.

The intra-assay and inter-assay coefficients of variation were < 10% and < 12%, respectively. Based on the manufacturer’s guidelines, a cut-off value of 250 pg/mL was used to distinguish between low (< 250 pg/mL) and normal (≥ 250 pg/mL) NKA.

### Statistics

Categorical variables are presented as counts with corresponding percentages, and continuous variables are summarized using median and interquartile range (IQR). In boxplots, the whiskers extend to the smallest and largest values within 1.5 times the IQR below the first quartile (Q1, 25th percentile) and above the third quartile (Q3, 75th percentile), respectively. Values outside this range are considered outliers and are included in statistical analyses but not visually depicted in boxplots. In addition, data were visualized using stacked bar charts and connected scatter plots. The Fisher’s exact test was used to compare categorical data, and *p* < 0.05 was considered statistically significant. All statistical analyses were calculated using STATA/BE18.0 (StataCorp LLC, TX, USA).

## Results

### Study population

**Figure 1:**
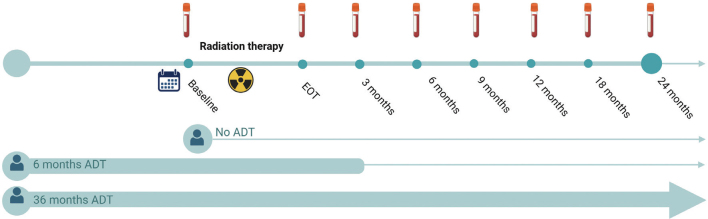
Overview of follow-up schedule and sample collection time points. The timeline illustrates the timing of RT, ADT initiation, and blood sample collection for NKA and PSA measurements. Figure created with BioRender.com

Among the 150 eligible patients, the median follow-up time at the time of data cut-off in May 2024 was 35 months (range, 5–57 months). For the present analyses, we focused on NKA dynamics during the first 24 months following RT. Blood samples were collected from all patients at BL, with subsequent sampling scheduled at the EOT and at follow-up (Fig. 1). The full 24-months follow-up data were available only for half of the cohort (Table 1). When focusing exclusively on the 2-year post-treatment period, 6 of 75 patients experienced recurrence, defined as either biochemical or radiological evidence of disease.

**Table 1:** Number of patients in each ADT group over time (*N* = 150).

	Baseline	EOT	3 months	6 months	9 months	12 months	18 months	24 months	Total
ADT treatment									
NO ADT	15	15	11	9	7	10	8	8	**83**
6 months ADT	23	20	18	19	16	15	15	14	**140**
36 months ADT	112	105	85	82	67	71	63	53	**638**
**Total**	**150**	**140**	**114**	**110**	**90**	**96**	**86**	**75**	**861**

EOT: End of treatment, ADT: Androgen deprivation therapy.

Baseline characteristics were compared between patients with normal and low NKA (Table 2). At baseline, the median NKA levels were 138 pg/mL (range: 0–247) for the low NKA group and 1,567 pg/mL (range: 263–20,000) for the normal NKA group. The overall median NKA was 832 pg/mL. Median baseline PSA levels were stratified by ADT group: 11 μg/mL (range: 5.4–18) in the no-ADT group, 2.4 μg/mL (range: 0.65–5.8) in the 6-month ADT group, and 1.85 μg/mL (range: 0–29) in the 36-month ADT group. As anticipated, baseline PSA values were lower in patients who had ADT before RT**.** Although most demographic and clinical parameters – including age, International Society of Urological Pathology (ISUP) grade, tumor (T) stage – were similar between the NKA groups (Table 1), smoking was more prevalent among patients with low NKA (27% vs. 11%). Additionally, 29% of the cohort had a body mass index (BMI) exceeding 30; however, BMI distribution did not differ significantly between the NKA groups (33% vs. 27%).

**Table 2:** Baseline characteristics, *N* = 150.

	Low NK cell activity *N* = 45 INF-γ < 250 pg/mL	Normal NK cell activity *N* = 105 INF-γ ≥ 250 pg/mL	Total *N* = 150
**Age, median (min;max)**	70 (53;77)	70 (53;78)	70 (53;78)
**Follow-up (months), median (min;max)**	31 (5;57)	35 (5;57)	35 (5;57)
**INF-γ, median (min;max)**	138 (0;247)	1556 (263;20,000)	831.5 (0;20,000)
**ADT treatment, *N* (%)**			
No ADT	7 (16)	8 (8)	15 (10)
6 months ADT	3 (7)	20 (19)	23 (15)
36 months ADT	35 (78)	77 (73)	112 (75)
**Performance status at diagnosis, *N* (%)**			
0	35 (78)	85 (81)	120 (80)
1	10 (22)	19 (18)	29 (19)
Unknown	0 (0)	1 (1)	1 (1)
**Risk group, *N* (%)**			
Low risk	0 (0)	1 (1)	1 (1)
Intermediate risk	9 (20)	27 (26)	36 (24)
High risk	36 (80)	77 (73)	113 (75)
**PSA**			
Diagnosis, median (min;max)	18 (4.1;121)	13 (2.5;97)	14 (2.5;121)
Baseline*, median (min;max)	3.3 (0.13;19)	2 (0;29)	2.3 (0;29)
**T-stage, clinical, *N* (%)**			
< T3	19 (42)	45 (43)	64 (43)
≥ T3	26 (58)	58 (56)	85 (57)
Tx/missing	0 (0)	1 (1)	1 (1)
**Lymph node metastasis**			
Yes	1 (2)	2 (2)	3 (2)
**ISUP, *N* (%)**			
Grade 1	2 (4)	2 (2)	4 (3)
Grade 2	18 (40)	34 (32)	52 (35)
Grade 3	11 (24)	39 (37)	50 (33)
Grade 4	6 (13)	10 (10)	16 (11)
Grade 5	8 (18)	20 (19)	28 (19)
**Smoking status, *N* (%)**			
Smoker	12 (27)	12 (11)	24 (16)
Non smoker	30 (67)	91 (87)	121 (81)
Missing	3 (7)	2 (2)	5 (3)
**BMI, *N* (%)**			
BMI < 30	29 (64)	73 (70)	102 (68)
BMI ≥ 30	15 (33)	28 (27)	43 (29)
Missing	1 (2)	4 (4)	5 (3)
**Autoimmune disease, *N* (%)**			
Yes	4 (9)	8 (8)	12 (8)
No	41 (91)	97 (92)	138 (92)

Percentages do not always add up to 100% due to data rounding.

* : Before initiating radiotherapy, NK cell: Natural killer cell, INF-γ: Interferon-gamma, ADT: Androgen deprivation therapy, PSA: Prostate-specific antigen, T-stage: Tumor stage, ISUP: International Society of Urological Pathology, BMI: Body mass index.

Distribution of normal and low NKA across ADT duration groups over time (categorical data)

**Figure 2:**
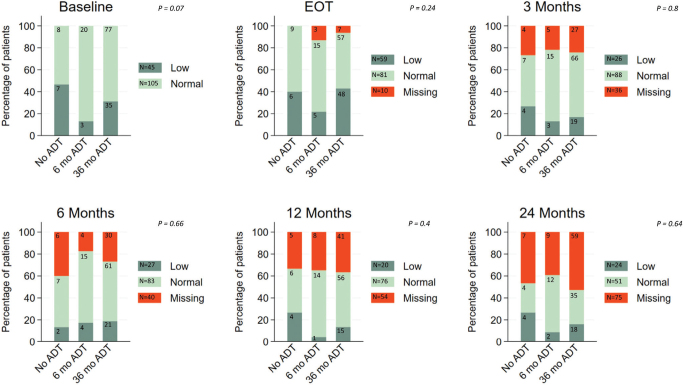
Distribution of NKA at baseline and follow-up timepoints among patients receiving no ADT, 6 months of ADT, or 36 months of ADT.

We investigated whether the distribution of patients with normal and low NKA differed between the three ADT groups. This was assessed at all selected time points up to 24 months post-RT (Fig. 2). Although no significant differences were observed in the distributions of low and normal NKA, there was a trend toward a higher frequency of low NKA at baseline in patients who did not receive ADT (*p* = 0.07). The number of observations decreased over time due to patients not completing the full 24-month follow-up, resulting in missing NKA measurements at later time points.

Distribution of normal and low NKA across ADT duration groups over time (continuous data)

**Figure 3:**
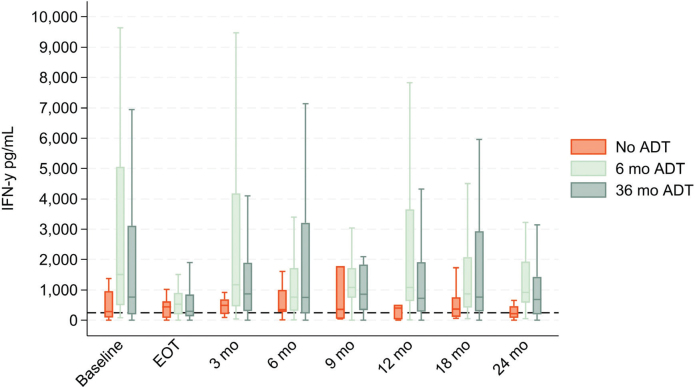
Boxplot displaying NKA in the three ADT groups at baseline, end of treatment (EOT), and at each follow-up. Outliers are not depicted but are included in the statistical analyses.

Considering NKA as a continues measure, we examined the change in NKA levels during the time course up to 24 months post-RT across the three ADT groups (Fig. 3). The number of patients with available NKA measurements at each time point, stratified by the ADT group, is presented in Table 1. The 6-month and 36-month ADT groups demonstrated the greatest variability in NKA over time. No consistent pattern in the variations was observed; however, in all cases, the median NKA level remained within the normal range above 250 pg/mL. In contrast, the No-ADT group showed more stable NKA levels with median values closer to the established 250 pg/mL IFN-γ cut-off at all time points. A marked reduction in IQR was observed at the EOT compared to BL in the two groups receiving ADT. For the 6-month ADT group, the median NKA at BL was 1502 pg/mL (IQR: 4559), decreasing to 535 pg/mL (IQR: 697) at EOT. Similarly, in the 36-month ADT group, the median NKA dropped from 765 pg/mL (IQR: 2914) at BL to 297 pg/mL (IQR: 714) at EOT. Conversely, in the no-ADT group, NKA levels increased at EOT compared to baseline. The median at BL was 284 pg/mL (IQR: 861), rising to 441 pg/mL (IQR: 539) at EOT.

### Individual variation in NKA and PSA

**Figure 4:**
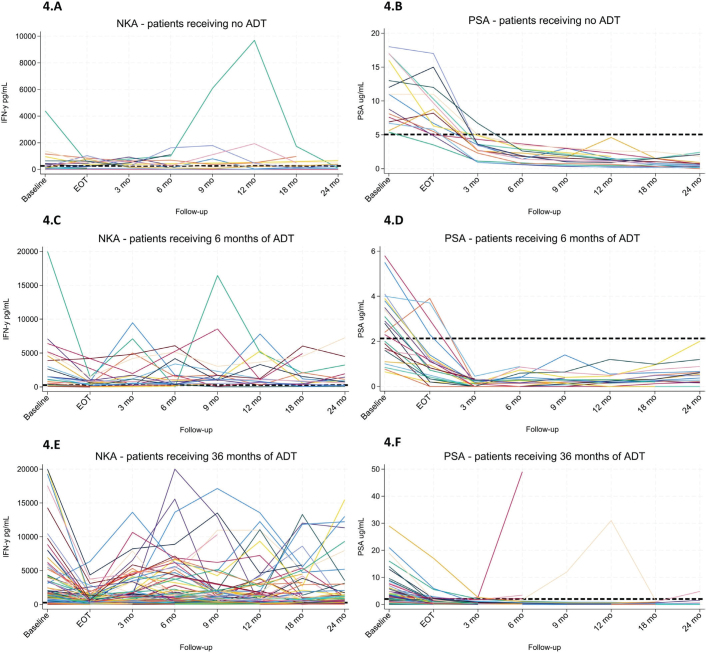
(A–F) Time-course profiles of natural killer cell activity (NKA) and prostate-specific antigen (PSA) in patients receiving no ADT (A–B), 6 months of ADT (C–D), or 36 months of ADT (E–F). In the NKA plots (A, C, E), the dotted horizontal line indicates the cut-off value for normal activity (250 pg/mL). In the PSA plots (B, D, F), the dotted horizontal line represents a reference threshold for potential biochemical recurrence, defined as the group-specific median PSA at 3 months post-treatment plus 2 ng/mL.

To obtain more insight into the variation of NKA, a detailed longitudinal analysis was conducted for each individual patient (Fig. 4, A, C, E). Patients in the 6-month and 36-month ADT groups exhibited a uniform decline in NKA from BL to EOT; however, post-treatment trajectories varied widely, with some patients displaying marked fluctuations, including one case exceeding 20,000 pg/mL. Among the 114 patients with available NKA measurements at both BL and 3-month follow-up, 15 (13.2%) had low NKA at both time points (low/low), 12 (10.5%) shifted from low to normal (low/normal), 11 (9.6%) from normal to low (normal/low), and 76 (66.7%) maintained normal levels at both time points (normal/normal). In contrast, PSA levels followed a more consistent pattern across all ADT groups, with a sharp decline from BL to 3 months post-treatment, followed by a gradual plateau (Fig. 4B, D, F).

Patterns of NKA and PSA dynamics in patients with biochemical or radiographic recurrence

**Figure 5:**
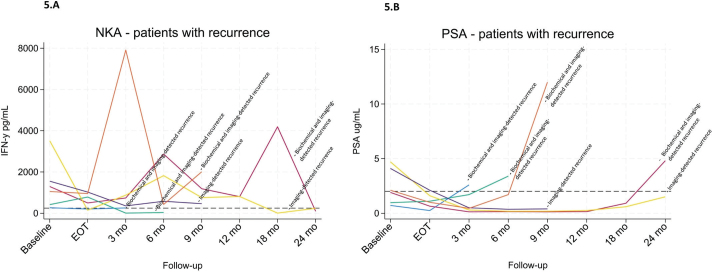
(A–B) Longitudinal changes in NKA (A) and PSA (B) in six patients who experienced biochemical and/or radiographic recurrence within 24 months. Case 1 (blue), Case 2 (red), Case 3 (green), Case 4 (yellow), Case 5 (purple), and Case 6 (orange).

Longitudinal changes in NKA and PSA were examined in patients who experienced biochemical or radiographic recurrence within 24 months of follow-up (Fig. 5). Of the 75 patients with complete 24-month follow-up, 8% developed recurrence, all of whom belonged to the group originally scheduled to receive 36 months of ADT. However, one of these patients discontinued treatment after only 6 months due to unacceptable side effects. Four of the six patients exhibited a PSA increase consistent with biochemical recurrence, while all six showed evidence of radiographic recurrence. Five of the six patients displayed either persistently low NKA or marked declines in NKA preceding recurrence. Individual patterns varied: Case 1 demonstrated persistently low NKA throughout follow-up, with recurrence confirmed both biochemically and radiologically. Case 2 exhibited a sharp decline to low NKA levels not previously observed in this patient at the time of recurrence, which was confirmed by both PSA rise and imaging. Case 3 showed a drop to low NKA at 3 months, which persisted at 6 months when recurrence was detected through biochemical and radiological evaluation. Case 4 experienced a decline to low NKA at 18 months, sustained at 24 months, where recurrence was identified on imaging alone. Case 5 demonstrated a gradual decline in NKA to near the assay cut-off between 3 and 9 months, coinciding with recurrence detected radiologically in the lung; notably, this case did not exhibit a concurrent rise in PSA. Case 6 deviated from the overall pattern, maintaining high NKA levels despite the most pronounced PSA increase in the cohort, with recurrence confirmed both biochemically and radiologically.

## Discussion and conclusion

This study presents novel data on the dynamics of peripheral NKA in patients with PCa treated with RT ± ADT. We report substantial variability in NKA, both across patients and over time, highlighting the complexity of interpreting immune activity in this context.

The heterogeneous population of NK cells that vary across different tissues must be taken into consideration when investigating NKA in different patient populations, across various tumor types, and under different treatment modalities.

Patients with localized and locally advanced PCa also present with a heterogeneous disease profile, and treatment strategies vary across risk groups. The risk of recurrence following RT is around 25–50% after 5 years [25–28], and the role of the immune system in this process remains unclear.

The mechanisms by which RT induces tumor hypoxia, affects NK cells, and modulates the tumor microenvironment are complex and not yet fully understood [19, 20]. NK cells, however, are shown to enhance the efficacy of RT by promoting antitumor immune responses. In our study, we observed a marked reduction in NKA following RT in patients treated with ADT, followed by a gradual recovery post-treatment. This recovery was accompanied by more variable fluctuations in NKA levels over time (Fig. 3). This pattern aligns with findings by Yamazaki et al. [29], who reported reduced NK activity after external RT, with or without brachytherapy, in patients with various solid tumors (*N* = 27). Similarly, McGinnes et al. found that the impact of RT on NKA was influenced by the irradiated site, with a reduction observed in patients receiving mediastinal irradiation [30].

Several potential interactions between NK cells and RT have been proposed, including RT-induced NK cell expansion and activation depending on dose and fractionation as well as modulation of NK cell function based on the irradiation [31]. These findings support growing interest in combining RT with NK cell-targeted therapies to enhance antitumor responses.

Interestingly, the highest baseline NKA levels were seen in patients receiving ADT supporting observations from other studies [18, 19]. While ADT suppresses tumor growth primarily via androgen depletion, it may, in turn, also exert immunomodulatory effects, such as promoting tumor reoxygenation, dampen release of immune modulating factors from the tumor, and enhancing immune cell infiltration [15]. The immune-enhancing properties of ADT, together with its associated improvement in tumor control, may account for the elevated baseline NKA observed in these patients.

NKA levels in our cohort were generally higher than those reported in other cancer types. The overall median NKA was 831.25 pg/mL (range: 0–20,000 pg/mL). This contrasts with the findings of Lu et al., who reported a median of 484.66 pg/mL (range: 246.32–818.70 pg/mL) in patients undergoing prostatectomy [10]. However, that group did not receive ADT. Variations in both the cohort composition and treatment and sampling methods may account for the observed difference. Moreover, studies in lung [32], recurrent ovarian cancer [33], and colorectal cancer [34] have reported considerably lower median baseline NKA levels (158, 131, and 86 pg/mL, respectively), likely reflecting more advanced disease stages or an entirely different underlying biology across cancer types.

The exceptionally high levels of NKA observed in some patients may reflect ADT treatment, age [35], and underlying tumor biology. Comparable findings may have been observed in other studies; however, as the only other study resembling ours in terms of both sampling- and incubation technique did not report NKA levels above 1,000 pg/mL, this remains uncertain [7].

There is no consensus on the optimal cut-off for defining low NKA in PCa. Our study employed a threshold of 250 pg/mL, as recommended by the assay manufacturer. However, thresholds of 200 pg/mL [6, 7, 11] and 500 pg/mL [8] have also been used, suggesting that tumor-specific or context-dependent criteria may be necessary. Binary classification may aid clinical interpretation, but risks oversimplifying a biologically continuous variable. Future studies should evaluate whether stratified or continuous NKA metrics provide greater prognostic or predictive value in PCa treated with RT ± ADT.

Lifestyle and comorbid factors such as smoking, obesity, and immunosuppressive treatments can influence NKA and may contribute to the observed variability. In our cohort, current smoking was more prevalent among patients with low NKA (27% vs. 11%), consistent with findings by Jung et al. [36], who reported immune suppression linked to tobacco exposure. This highlights that cigarette smoking may disrupt immune defenses, promoting the ability of cancer cells to evade immune detection and elimination [36]. It also indicates that NKA, as a biomarker, may be affected by confounding factors.

Almost one-third of the patients in our cohort were classified as obese. Obesity may impair NK cell function and longevity [37], and physical inactivity and unhealthy metabolic status have been associated with decreased NKA [38]. The relationship is complex, as a high BMI also has been associated with increased activation of peripheral NK cells, yet also with a more dysfunctional immune response, which may help explain the increased susceptibility to cancer and infectious diseases in individuals with obesity [39].

NKA may be involved in the pathogenesis of immune-mediated diseases [40], and immunosuppressive drugs may exert a suppressive effect on NK cells [41]. We examined the influence of autoimmune diseases and immunosuppressive medications on NKA in patients with PCa but found no consistent pattern. The lack of association may reflect multifactorial regulation of NK cell function, in which pharmacological immunosuppression plays a relatively minor role.

Only six patients experienced disease recurrence, limiting the statistical power to evaluate NKA as a prognostic marker. Although recurrence events were infrequent, five of six patient cases demonstrated either persistently low or markedly declining NKA levels preceding recurrence, suggesting a potential association. Prior studies have reported that low NKA correlates with poorer prognosis in various malignancies, including PCa [10, 32, 42, 43]. Interestingly, in metastatic PCa, higher NK cell cytotoxicity has been associated with improved outcomes such as delayed castration resistance [43]. However, other findings in metastatic castration-resistant PCa (mCRPC) suggest that elevated NKA may paradoxically predict poorer treatment response [44], underscoring the need to account for disease stage and treatment context when interpreting NKA levels.

Several limitations should be acknowledged. First, the low number of recurrence events limited our ability to perform prognostic and multivariable analyses. Second, incomplete biomarker follow-up in some patients, particularly at later time points, caused missing data and reduced the availability of longitudinal data. Third, a 24-month follow-up may not fully capture long-term oncological outcomes, particularly in a curative-intent setting. Finally, the design of the study does not allow for separation of the independent effects of ADT and RT on NKA, as the BL measurement was obtained after the initiation of ADT but before RT for most of the cohort. The small size of the no-ADT group (*n* = 15) limits the statistical power, and the estimates may, therefore, be interpreted with this uncertainty in mind. In this context, a sample collected at diagnosis would have strengthened the study by allowing a clearer interpretation of the effect of ADT on NKA.

Although we observed a possible association between declining or persistently low NKA levels and disease recurrence in a small subset of patients, our overall findings highlight the complexity of NKA as a biomarker. Multiple influencing factors and pronounced inter- and intra-patient heterogeneity suggest that NKA may have limited reliability in patients with localized or locally advanced PCa treated with RT.

The present cohort will continue to be followed to determine whether early or persistent suppression of NKA correlates with long-term oncological outcomes, and a larger validation cohort from the same population is being assembled. Future analyses will explore the immunophenotype of patients with consistently low or declining NKA, providing insight into the role of innate immunity in PCa progression. It will also be relevant to investigate the interplay among testosterone levels, treatment parameters, and NKA dynamics.

## Conclusion

This prospective study examined NKA in patients with localized/locally advanced PCa undergoing curatively intended RT with or without (neo) adjuvant ADT. NKA levels showed substantial inter-patient variability and fluctuated during treatment and follow-up. Current smoking was significantly associated with reduced baseline NKA. Patients receiving ADT had higher pre-RT NKA and a pronounced decline at RT completion, with partial recovery during follow-up. Some patients exhibited unexpectedly elevated and fluctuating NKA levels, which did not parallel PSA dynamics. Although some cases of biochemical recurrence coincided with declining NKA, the low event rate limits definitive conclusions. The overall complexity and heterogeneity of NKA raise questions about its clinical utility as a biomarker in this setting. Further research is warranted to clarify the potential role of NKA as a biomarker in patients with localized/locally advanced PCa.

## Data Availability

The data that support the findings of this study are not publicly available due to restrictions related to patient confidentiality and privacy. Access to the data may be available upon reasonable request and with approval from the appropriate ethics committee.
